# A Clinical Text-Book of Medical Diagnosis for Physicians and Surgeons, Based on the Most Recent Methods of Examination

**Published:** 1899-07

**Authors:** 


A Clinical Text-Book of Medical Diagnosis for physicians and surgeons, based on the most recent methods of examination. By Oswald Vierordt, M. D., Professor of Medicine at Hiedelberg, etc. Authorized translation with additions by Francis H. Stuart, A. M., M. D., Member of the Medical Society of the County of Kings, Fellow of the New York Academy of Medicine, etc. Fourth American edition from Fifth German. Published by W. B. Sanders, Philadelphia. Pages, 600. Price, cloth, $4.00; leather, $5.00.

This truly great work comes to us again, greatly improved. It might be truly said that medical progress consists almost entirely in the progress made in the science of diagnosis. This being true, it can be readily seen that it is absolutely necessary that the progressive physician should have the latest and best work on diagnosis. I remember that my teacher in anatomy once said to his graduating class, holding in his hand the latest edition of "Gray": "Gentlemen, here is a book not for your library, but for your desk or table." The same can be said of our work on diagnosis. It should not be stored in the book-case, but kept constantly before our eyes.

The fact that five editions and four translations have been called for in eight years, sufficiently attests the worth and popularity of the work. It consists, exclusive of introduction and appendix, of five chapters on the following subjects: General examination, examination of the respiratory apparatus, examination of the circulatory apparatus, examination of the digestive apparatus, examination of the urinary apparatus and examination of the nervous system. The appendix consists of concise and practical articles upon laryngoscopy, rhinoscopy, otoscopy, optholmoscopy and bacteria, which are to be considered in the diagnosis of internal disease. The work is covered by a thorough index; indeed, I have searched it for the purpose of seeking out all the newer diagnostic methods, bacteria, etc., and have not found it at fault in a single instance. J.

